# Management of urachal cancer in pregnancy: A systematic review

**DOI:** 10.1002/bco2.170

**Published:** 2022-05-29

**Authors:** Kenneth K. Y. Chew, Samantha Quah, Madison R. Boot, Steven Sowter

**Affiliations:** ^1^ University of New South Wales Sydney New South Wales Australia; ^2^ Wagga Wagga Base Hospital Wagga Wagga New South Wales Australia; ^3^ University of Sydney Sydney New South Wales Australia

**Keywords:** management, pregnancy, systematic review, urachal cancer, urology

## Abstract

Urachal cancer is a rare non‐urothelial malignancy that involves the urachus, often occurring at the junction of the urachal ligament and the bladder dome. It accounts for less than 1% of all bladder tumours. Cancer during pregnancy is rare, with the incidence of all cancers in pregnancy estimated to be 25–27 per 100 000 pregnancies. Urachal cancer in pregnancy is an even rarer phenomenon, with only a handful of case reports published to date. After a systematic review, only five cases have been reported in the English literature. We aim to review the cases presented in the literature and to examine the outcomes of the management of urachal cancer in pregnancy to date.

## 1 INTRODUCTION

Urachal cancer is a rare non‐urothelial malignancy that involves the urachus, often occurring at the junction of the urachal ligament and the bladder dome. Urachal tumours display adenocarcinoma histology and are believed to originate from residual enteric rests during embryological development or from metaplastic change of the urachus.^1^ It accounts for less than 1% of all bladder tumours.^2^ To date, the most comprehensive data we have on urachal cancer is from Szarvas et al. who conducted a meta‐analysis of 1000 cases of urachal cancers.^3^ This review demonstrated that the median age of diagnosis was 52 years, that it was more frequent in men (59%), and that the most common symptoms were haematuria (73%), abdominal pain (14%) and mucosuria (10%).^3^

Historically, the prognosis of urachal cancer was reported as dire due to its aggressive nature and diagnosis at advanced stages, with up to 21% of patients with primary metastatic disease at time of diagnosis.^3^ However, in a recent review of 46 cases by Dhillon et al., it was demonstrated that the median survival time was 45 months, which was significantly longer than patients with bladder urothelial carcinoma of similar‐stage disease.^4^ Szarvas et al. also demonstrated a 50% 5‐year overall survival rates in patients with Sheldon staging Stage IIIA and below (Table 1).^5^ Mayo staging was also found to be superior in predicting survival rates, with a Mayo stage less than II being associated with shorter survival (Table 2).^6^, ^7^

**TABLE 1:** Sheldon staging system for urachal cancer

Sheldon staging	Findings
Stage I	Urachal cancer confined to urachal mucosa
Stage II	Urachal cancer with invasion confined to urachus
Stage IIIA	Local urachal cancer extension to bladder
Stage IIIB	Local urachal cancer extension to abdominal wall
Stage IIIC	Local urachal cancer extension to peritoneum
Stage IIID	Local urachal cancer extension to viscera other than bladder
Stage IVA	Metastasis to regional lymph node
Stage IVB	Metastatic urachal cancer to distant sites

*Note*: Table reproduced from Sheldon et al.^5^

**TABLE 2:** Mayo staging system for urachal cancer

Mayo staging	Findings
Stage I	Tumour confined to urachus and/or bladder
Stage II	Tumour extending beyond the muscular layer of urachus and/or the bladder
Stage III	Tumour infiltrating regional lymph nodes
Stage IV	Tumour infiltrating non‐regional lymph nodes or other distant sites

*Note*: Table reproduced from Henly et al.^8^

Cancer during pregnancy is rare, with the incidence of all cancers in pregnancy estimated to be 25–27 per 100 000 pregnancies.^9^ Urachal cancer in pregnancy is an even rarer phenomenon, with only a handful of case reports published to date. After a systematic review, only five cases have been reported in the English literature. We aim to review the cases presented in the literature and to examine the outcomes of the management of urachal cancer in pregnancy to date.

## 2 METHODOLOGY

This review was performed according to the preferred reporting items for systematic reviews and meta‐analyses (PRISMA) statement and PRISMA checklist. Three databases were searched including Pubmed, Medline, and Embase. The search was conducted on the articles of the last with no year restrictions. Search terms included were [‘urachal cancer’ OR ‘urachal mass’ AND ‘pregnancy’]. A language restriction to English papers was applied.

All age groups were included. Duplicate studies and non‐English studies were excluded. The abstracts reviewed by the four authors (K.C., S.Q., M.B. and S.S.) independently for inclusion and exclusion criteria. Disagreement resulted in exclusion of the articles. Articles that met the inclusion and exclusion criteria after the three independent authors review were included, the selection process outlined in Figure S1.

Data extracted include patient details, gestational age at diagnosis, method of diagnosis, termination of pregnancy status, management, and outcomes.

## 3 RESULTS

The average age was 34 and ranged from 28 to 42. The average gestational age at diagnosis was 21 weeks and ranged from 8 to 38 weeks. Two of the patients presented with gross haematuria,^10^, ^11^ one presented with a palpable umbilical mass and pain,^12^ and two patients had incidental findings of urachal cancer.^13^, ^14^ Importantly, regardless of presenting symptoms, four of the five patients had a bladder dome lesion or a lesion superior to and contiguous with the bladder dome demonstrated on abdominal ultrasound. All five patients underwent surgical resection. Four patients underwent partial cystectomy with urachal excision. Three patients also underwent radical umbilectomy. Four of the patients had good outcomes with disease‐free survival. One patient had a relapse of disease. Two patients underwent termination of pregnancy, the first one had presented for a termination of pregnancy and second had tumour extension into the uterus and obvious lymph node involvement at surgical excision.^10^, ^13^ Interestingly, four of the five patients were multiparous. However, little to no information has been provided by the authors regarding the previous pregnancies.

A summary of these cases will be provided in Table 3.

**TABLE 3:** Summary of case reports in the literature

Author (year)	Age	Gestational age at diagnosis (weeks)	Method of diagnosis	Sheldon stage	Termination of pregnancy	Treatment	Prognosis
Pasternak et al. (2014)	28	8	Request for termination of pregnancy, lesion superior to bladder demonstrated on US	I	Y	Surgery (Radical umbilectomy, urachal excision, partial cystectomy, omentectomy)	Alive, unknown time
McNally et al. (2013)	36	9	Abdominal pain, palpable umbilical mass, lesion superior to bladder demonstrated on US	IIIB/C	N	Surgery (Radical umbilectomy, urachal excision, partial cystectomy)	Alive at 16 months
Van Calsteren et al. (2006)	31	14	Gross haematuria, lesion in bladder dome demonstrated on US	IVA	Y	Surgery (Urachal excision, partial cystectomy), radiotherapy	Alive at 6 months with relapse, planned radiotherapy
Genel et al. (2020)	42	38	Gross haematuria with UTI, lesion in bladder dome demonstrated on US	IIA	N	Surgery (Radical cystectomy)	Alive, unknown time
Goldman et al. (2016)	35	38	Incidentally during caesarean section, lesion in uterus demonstrated on US	I	N	Surgery (Radical umbilectomy, urachal excision, partial cystectomy)	Alive at 6 months

## 4 DISCUSSION

Urachal cancer in pregnancy is rare but can be associated with poor outcomes if presenting with advanced disease regardless of pregnancy status, common given patients are often asymptomatic in initial stages. Therefore, early recognition and definitive treatment is paramount in obtaining good outcomes. Urachal cancer should be suspected in pregnant patients presenting with gross haematuria, abdominal pain or recurrent urinary tract infections which shows concordance with presentations in non‐pregnant patients.^3^, ^10^, ^11^, ^12^ A comprehensive physical examination is important and can reveal an abdominal mass separate from the gravid uterus in the umbilical region.^12^ Urine cytology has not been demonstrated to be effective in consistently detecting urachal cancer. Henly et al. demonstrated only 17% of their patients (*n* = 4/24) with urachal cancer had positive urine cytology, whereas Ashley et al. showed only 34% of their patients (*n* = 13/38) had positive urine cytology.^7^, ^8^ Thus far, there are no studies directly comparing the incidence of positive urine cytology and grade of cancer at diagnosis. However, it can be postulated that because urachal cancer is primarily an extra‐vesical disease, the presence of positive urine cytology heralds locally advanced disease when the tumour has invaded the bladder.

Imaging is an invaluable tool for diagnosis. The use of ultrasonography (US) is safe in pregnancy can reveal a supra‐vesical mass contiguous with the dome of bladder. The most common finding is a mass with low‐attenuation component with calcifications reflecting the mucinous content of the tumour.^15^ Computed tomography (CT) or magnetic resonance imaging (MRI) can aid in confirming US findings and provide additional information regarding the local extent of the tumour, regional lymph node involvement and metastatic disease. Classically, urachal tumours are said to be detectable and diagnosed due to the pathognomonic presence of calcifications which are demonstrated on both CT and MRI as areas of increased signal intensity.^16^, ^17^ In 2016, Szarvas et al. demonstrated on systematic review that only 32% of patients (*n* = 45/142) had CT findings of calcifications therefore the absence of calcification does not necessarily rule out urachal adenocarcinoma.^3^ Regardless, the detection of a supravesical mass contiguous with the bladder should elicit a high level of suspicion for a urachal tumour. The use of MRI in pregnancy is recommended as it eliminates radiation risk to the foetus whilst providing high rates for detection of a urachal mass. The other advantage of MRI is to detect lymph node involvement which will affect whether lymph node dissection is undertaken.

In addition to clinical examination and imaging, cystoscopy is the most important diagnostic tool for urachal cancer. In a systematic review done by Szarvas et al. in 2016, from 276 patients with data on cystoscopy, a positive finding was demonstrated in 245 of these patients (89%).^3^ Cystoscopy can provide tissue diagnosis, can aid in localizing the tumour and aid surgical planning for eventual surgical resection. In a 2020 review of the literature of the management of bladder tumours in the pregnant population (*n* = 47), it was shown that cystoscopy under regional or general anaesthesia is indicated and has been safely performed if suspecting bladder cancer regardless of gestational age.^18^ The safety of anaesthesia in pregnancy is always a concern for both clinician and patient and does not come without risk. Cohen‐Kerem et al. reported a 5.8% miscarriage rate in patients undergoing general anaesthesia in the first trimester.^19^ However, it is important to note that reported rates of miscarriage in healthy women can be as high as 26%.^20^ Importantly, secondary complications from disease progression must be considered when planning surgical management and delays may increase the risk to both mother and foetus if not promptly managed.^21^ Regardless, a multi‐disciplinary approach involving the urologist, anaesthetist and obstetrician to counsel the pregnant patient with urachal cancer regarding cystoscopy and surgery is advised.

The recommended treatment for nonmetastatic urachal cancer is surgery. Both partial and radical cystectomy provide comparable oncological results. As partial cystectomy provides a higher quality of life given its organ‐preserving nature, it should therefore be the preferred option.^7^, ^22^ Additionally, complete resection of the urachal remnant and radical umbilectomy is essential for longer term survival and can provide long‐term disease‐free survival in patients with localized disease. Surgery can be performed at any stage of pregnancy and should be offered to patients if there is a high index of suspicion for urachal cancer.^23^ Patients should be counselled that if metastatic disease is detected prior to or encountered during surgical resection, termination of pregnancy may be advised in order to achieve surgical margins and to expedite management with adjuvant chemotherapy.

Systemic chemotherapy in urachal cancer is mainly empirical due to the lack of randomized studies in such a rare malignancy. Siefker‐Radtke et al. have demonstrated that the highest response rates in patients receiving 5‐fluorouracil (5‐FU)‐based and cisplatin‐based therapies with a combination of the two providing the most favourable response in metastatic urachal cancer, with a suggestion of 30–40% radiographic response rates.^24^ There is no currently available evidence of improved survival outcomes in the use of chemotherapy in metastatic urachal cancer. In regard to the safety of systemic chemotherapy in pregnancy, the use of both the use of both cisplatin and 5‐FU has been shown to be safe for use during the second and third trimesters of pregnancy in various systemic reviews of the use of chemotherapy in pregnant patients with breast and ovarian cancer.^25^, ^26^ However, there is a paucity of research regarding the administration of chemotherapy during the first trimester of pregnancy, with case reports or case series making up the bulk of the evidence. In general, it is well‐recognized that the safety of chemotherapy agents is directly correlated with gestational age. In 2016, a non‐systematic review by Miyamoto et al. demonstrated a large percentage of foetal abnormalities in rat models having received 5‐FU in the first trimester with up to 30% of the subjects developing major abnormalities.^16^ Congenital foetal abnormalities have also been reported in a case report in which 5‐FU was administered in the first trimester.^27^ The safety of cisplatin has also been demonstrated to be harmful causing major foetal abnormalities when administered in the first trimester.^28^ Systemic chemotherapy is a safe option in the second and third trimester of pregnancy. However, with no evidence of improved survival outcomes, the benefits of chemotherapy must be weighed up against the side effects of chemotherapy.

It is important to state that uncertainty exists regarding the junction between second and third trimester pregnancies, on whether to perform surgical resection with risk of premature delivery or miscarriage versus delay in treatment leading to progression of disease. Ultimately, the decision is a shared process between the patient and their family, the urologist and the obstetrician.

## 5 CONCLUSION

In summary, urachal cancer is a rare occurrence in pregnancy but can pose a management conundrum even for the most experienced urologists. Urachal cancer is an aggressive form of cancer that needs to be promptly diagnosed and managed, preferably with a multi‐disciplinary team. Pregnancy should not be a contraindication to patients undergoing radical operative management aiming for curative intent for urachal cancer. Surgery during all trimesters can be performed safely, whereas systemic chemotherapy can be safely administered in the second or third trimester, although this has not been proven to improve outcomes. More prospective data are required to determine optimal chemotherapy regimens and outcomes of treatment of urachal cancer specifically in pregnancy, perhaps in the form of a registry.

## CONFLICT OF INTERESTS

No potential competing interest was reported by the authors.

## Supporting information


**Figure S1.** PRISMA flow diagram
