# Supplementary material for: Management of urachal cancer in pregnancy: A systematic review
Source: BJUI Compass. 2022 May 29;3(6):410–4. doi: 10.1002/bco2.170 (PMC9579884; doi:10.1002/bco2.170)
Supplement: Supplementary file 1 — Figure S1. PRISMA flow diagram [file BCO2-3-410-s001.docx]

**Identification of studies via databases PUBMED, MEDLINE, EMBASE and OTHER**

Records identified from PUBMED

(n = 19)

**Identification**

Duplicate records excluded

(n = 4)

Articles collated after search

(n = 20)

Records after duplicates removed

(n = 16)

**Eligibility**

Records screened based on title and abstract

(n = 6)

Full text articles assessed for eligibility

(n = 6)

Articles included in final review

(n = 5)

**Included**

**Screening**

Records excluded

(n =10)

Records excluded

(n = 1)

1.Non-English
